# An Address Delivered before the Pennsylvania Society of Dental Surgery

**Published:** 1854-01

**Authors:** 


*An address delivered before the Pennsylvania Society of Dental Surgery, by* ELISHA TOWNSEND, D. D. S., *etc. Opening address delivered before the Society of the Alumni of the Baltimore College of Dental Surgery, by* E. TOWNSEND, D. D. S.

WE are under obligations to Dr. ELDER for a copy of each of the above productions.

Dentistry has already become a science as well as an art. Some of the best minds in our country are engaged in the study and practice of it, correcting irregularities and repairing deficiencies in the teeth, by applying the known laws of physiological science. An interesting feature in the history of Dentistry at the present day is the establishment of Colleges of Dental Surgery, where a regular and systematic course of study can be pursued, when the young man is taught not only how to extract and perform other mechanical operations on the teeth, but the anatomy of the strictures with which he has to deal; the relation they bear to other near and remote parts of the system; the diseases to which they are subject, their therapeutical indications, and the remedies appropriate for removing them. The superiority of the dentist thus educated over the mere tooth extractor is of the same character as that of the surgeon of the present day, well instructed in the art and skilled in the science of his profession, over the barber, whose only merit consists in knowing how to bleed, and leech, and cup.

Dental like general surgery, to accomplish its highest mission, must be conservative, it must seek not to mutilate but to save from mutilation. In reference to this subject, Dr. TOWNSEND very appropriately remarks:

The Dental Surgeon should remember that every time he extracts a tooth, he acknowledges against himself, or against his profession, or against both together, that he cannot cure, and therefore must mutilate. I repeat, it is the imperfection of our art, that we must extract at all.

It may be, indeed, in the very nature of things, impossible to avoid it in any conceivable state of knowlege; still that necessity will ever stand as a professional defect, and all progress, consists in diminshing that necessity. I am not ashamed of my workmanship, nor do I refuse the credit it gives me, but the man who will teach me how to save a tooth, that I am now obliged to sacrifice, is my master in the science of Dentistry, without the proof of any other claim, and I gladly yield him the post of honor.

It is in this regard, in the preservation, the cure, the prevention of disease, that general and thorough medical science becomes available, and takes the rank I would assign it in Dental Surgery.

The teeth are a part, and no mean or very remote part of the general organization, their sensibility is delicate, though subdued, and their liability to disease, *itself* indicates their acute activity of life. In the proportion of this sensibility and liability, as well as of their functional importance, they are interlinked by active sympathies with the entire frame. Disease in distantly situated, but nearly related organs, affects them symptomatically, and their original derangements react again upon associated organs. The entire digestive apparatus is involved in these connections, by direct functional relation. Dyspepsia has been unsuccessfully treated, and teeth have been erroneously and needlessly extracted, in ignorance or neglect of this mutual dependency. Indeed, the man who has not remedied tooth-ache by emetics, cathartics, and other medicinal appliances, to the primæ viæ, has either had but little practice in dentistry, or deserved to have still less. I have taken an obvious instance to represent a principle.

There are many less familiar connections, and many, doubtless, quite unknown, in which general science would prove available in our branch of remedial practice. Besides, idiopathic affections of the teeth and gums, are themselves diseases, and their treatment supposes an acquaintance with the laws of life, both healthy and abnormal, and with hygienic and therapeutic principles. I would have every Dentist go in debt to the other branches of medicine for all they can confer upon him, and than repay it all by some worthy contribution of discovery in remedial treatment to the general stock.

Did our limits permit, we should be glad to make still further extracts. The above is sufficient to show the liberal spirit with which this department of professional labor is at the present day pursued, and the earnestness with which the distinguished author of those addresses is endeavoring to advance the interest and elevate the standard of attainments in dental surgery. J.

